# Supplementary material for: Comparative Whole-Genome Sequence Analyses of Fusarium Wilt Pathogen (Foc R1, STR4 and TR4) Infecting Cavendish (AAA) Bananas in India, with a Special Emphasis on Pathogenicity Mechanisms
Source: J Fungi (Basel). 2021 Aug 31;7(9):717. doi: 10.3390/jof7090717 (PMC8469521; doi:10.3390/jof7090717)
Supplement: Supplementary file 1 [file jof-07-00717-s001.zip › jof-1323046-supplementary/jof-1323046-Supplementary Figure.pdf]

SNPs and InDels in the *Foc* Genome of Indian races

In general, the number of heterozygous are more than the homozygous SNPs in the *Foc* genome (Supplementary Table S1). Among the genome, a higher number of SNPs was observed in STR4 in which 1,681 are heterozygous SNPs and 163 are heterozygous, specifically, A (966) based SNPs followed by C (757) and G (121). The distribution of the total number of SNPs is relatively on par with the *Foc* R1 (1502) and TR4 (1520). However, the variation was observed in T based SNPs where in *Foc* R1 it is 316 and in TR4 it is 334 and the remaining subgroups are relatively the same. In contrast, the number of InDels are rich in *Foc* R1 (53,602) and TR4 (52,123) when compared to STR4 (33,298) in which InDels are higher in homozygous regions (1,32,054) and lower in heterozygous (6969) (Supplementary Table S2). Similarly, the insertions (88,532) are higher than the deletions (50,491) where the highest number of insertions were observed in *Foc* R1 (32,438) and TR4 (32,494) but it is minimum in STR4 (23,600). The deletions are common in the *Foc* R1 (21,164), followed by TR4 (19,629) and STR4 (9698). A higher number of SNPs were occurred in the intergenic region (1949) followed by non-synonymous (697) and synonymous coding regions (959) in which the maximum number of SNPs were the substitution of A in the intergenic region followed by C in the synonymous coding region and A in the non-synonymous coding region of heterozygotic origin (Supplementary Table S1&S2).

A

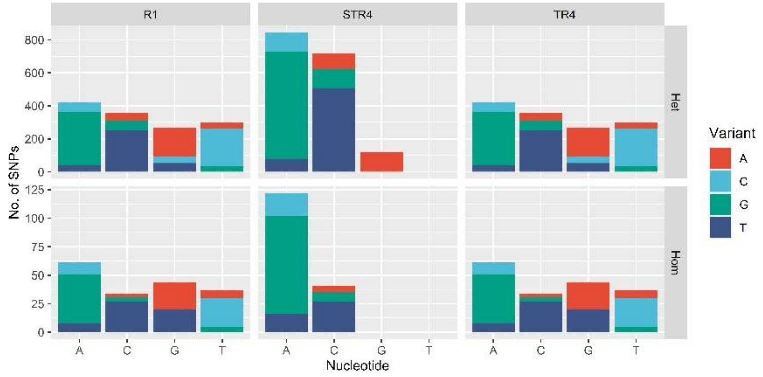

B

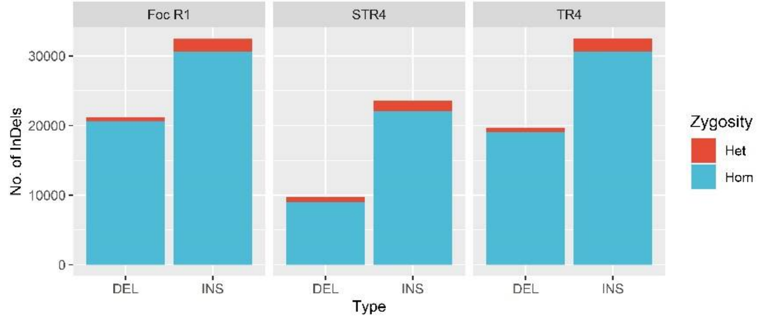

Supplementary Figure S1: Comparison of SNPs (**A**) and InDels (**B**) in the Indian Foc races. The legends represents the number of SNPs variation types based on nts bp (top), and types of Zygosity InDels observed in *Foc* genome.
